# Hyperlipidemia and Hypertension Are Associated With Intracerebral Hemorrhage Incidence: A Retrospective Study

**DOI:** 10.7759/cureus.33236

**Published:** 2023-01-01

**Authors:** Awad M Almuklass, Yousef A Alawad, Abdulmalek S Alanazi, Azzam A Alamro, Faisal H Alagedi, Yasser A Alshehri, Emad Masuadi, Naser Alotaibi, Mahmoud Alkhateeb

**Affiliations:** 1 Basic Medical Sciences, King Saud Bin Abdulaziz University for Health Sciences, College of Medicine, Riyadh, SAU; 2 Research, King Abdullah International Medical Research Center, Riyadh, SAU; 3 Public Health Institute, United Arab Emirates University, Al Ain, ARE; 4 Neurology and Internal Medicine, King Abdulaziz Medical City, Ministry of National Guard Health Affairs, Riyadh, SAU

**Keywords:** hypertension, hyperlipidemia, ischemic stroke, hemorrhagic stroke, stroke

## Abstract

Introduction

Stroke places a huge burden on the socioeconomic systems. Hemorrhagic stroke (HS) is the second most common type of stroke and the second leading cause of disability and death. The updated data on the prevalence of intracerebral hemorrhage (ICH) stroke and related physiological risk factors in Saudi Arabia were limited. The aim of this study was to identify the prevalence of ICH stroke and the related physiological risk factors.

Methods

This was a retrospective, hospital-based, and chart review study that utilized the BESTCare system at King Abdulaziz Medical City (KAMC), Riyadh, Saudi Arabia. Patients who attended the neurology department (inpatient/outpatient) between 2015 and 2020 were studied. The statistical tool JMP (JMP Inc., Cary, NC, USA) was used for data entry and analysis.

Results

Patient data (N = 1,870, 58.6 ± 13.87 years old) were screened for comorbidities, hypertension (66.1%), diabetes mellitus (DM) (57.7%), hyperlipidemia (28.4%), and history of an old stroke (22.3%). Ischemic stroke (IS) was more dominant than ICH stroke with ratios of 94.5% (n = 1767) versus 5.5% (n = 103), respectively. The prevalence of ICH stroke among the patients (n = 103) was 10.6%, 20.3%, 24.2%, and 28.1% in the age groups of <40, 41-50, 51-60, and 61-70 years old, respectively. There was a significant gender effect on the distribution of both IS and ICH (p = 0.003). ICH strokes were more prevalent in males than in females. Body mass index (BMI) has no significant effect on the prevalence of IS and ICH stroke (p = 0.081). ICH stroke was significantly associated with DM (p = 0.032), hypertension (p = 0.01), and hyperlipidemia (p = 0.002). Regression analyses show that only hypertension (positive association) and hyperlipidemia (negative association) were significantly associated with the incidence of ICH stroke.

Conclusion

IS was more prevalent than ICH stroke. ICH strokes were more prevalent in males than in females. Also, hypertension was the most common factor leading to ICH stroke, unlike hyperlipidemia, which was revealed to be protective against ICH stroke.

## Introduction

Hemorrhagic stroke is the second leading cause of global disability and death and the second most common type of stroke, accounting for 15%-20% of all stroke cases [1]. Blood vessel rupture in response to hypertension, cerebral amyloid deposits, aneurysms, and trauma all can lead to hemorrhagic stroke. Symptoms include headaches, sensory and motor issues (i.e., central vertigo, numbness, and sudden unilateral weakness), visual disturbances (i.e., diplopia), and speech abnormalities (i.e., aphasia and speech impairment) [2]. Although some occur as a subarachnoid hemorrhage in the brain membrane, 85%-95% of hemorrhagic strokes are caused by intracerebral hemorrhage (ICH) in the brain parenchyma [3], of which more than half (50%-75%) are deep supratentorial ICHs in the basal ganglia [4]. In recent years, the global prevalence of ICH stroke and associated disability-adjusted mortality and life-year rates have risen dramatically [5]. Reducing the burden of hemorrhagic stroke is an international priority that requires controlled research to determine key risk factors among different populations in low- and high-income countries [5]. This retrospective, hospital-based study thus aimed to identify the prevalence of ICH stroke and common physiological and pathophysiological risk factors in a Saudi population.

Several large-scale, epidemiological, clinical, and meta-analytical studies have been conducted in recent decades to explore common risk factors associated with ICH stroke. For example, the INTERSTROKE study is an international, multicenter, case-control study involving patients in 22 countries and assessing stroke by age group, gender, smoking habit, hypertension history, alcohol consumption, diet, and waist-to-hip ratio (obesity) [2]. Its results show that hypercholesterolemia is inversely associated with the risk of ICH and subarachnoid hemorrhage [3]. The 2019 Global Burden of Disease study found that ischemic stroke (IS), ICH, and subarachnoid hemorrhage accounted for 64.9%, 26.2%, and 8.9% of global stroke cases, respectively [5]. In the USA, Australia, and the UK, the prevalence of ICH stroke ranges from 8% to 15% [6], compared to 18% to 24% in Japan and Korea [7,8]. The incidence of ICH stroke is twice as high in high-income countries [3,9,10], higher among men than among women, and increases with age [11].

In the Middle East region, the prevalence of ICH stroke ranges from 7% to 37.8% [12]. In Saudi Arabia, one of the largest nations in the Middle East with a population of about 32 million (63% of whom are Saudi), stroke is the second leading cause of mortality and disability [13]. Total stroke incidence ranges widely from 15.9% to 57.6% across the Kingdom [14-18], and the number of stroke cases in Saudi Arabia is anticipated to double by 2030, in part due to cultural and social advancement [13]. However, recently published statistics indicate a low awareness of stroke and its risk factors among Saudis [14]. Stroke rates in Saudi Arabia also remain lower than those in the USA and UK, which may be attributable to the large proportion of younger individuals in the Saudi population [15]. However, most research on stroke prevalence among Saudi populations dates from 1991 to 1998, when the prevalence of ICH stroke ranged from 11% to 41.5% [16].

## Materials and methods

In this retrospective, hospital-based study, a cross-sectional research design was used to gather data from the neurology department of King Abdulaziz Medical City (KAMC) hospital in Riyadh (central province), the capital of the Kingdom of Saudi Arabia. This department contains 40 beds for critical stroke patients and sees at least 300 stroke patients each year. The KAMC neurology department comprises eight expert consultants with diverse specializations, such as stroke, sleep, neuromuscular, and movement disorders, as well as nine residents.

The study participant comprised 1,870 Saudi patients (1,219 males and 651 females) aged 58.6 ± 13.87 years who were admitted to the emergency department of KAMC between January 2015 and January 2020. Only patients admitted to the KAMC neurology unit were included in our study; patients admitted to any other unit were excluded. Patient data were obtained from the hospital information system and included the time and mode of arrival, medical history provided at the initial assessment, demographics (gender, age, height, weight, body mass index (BMI), and smoking history), clinical features (e.g., blood pressure on arrival), laboratory results (lipid profile, fasting blood glucose level, hemoglobin A1c (HbA1c), serum levels of low-density lipoproteins (LDL), type of stroke (ischemic versus ICH), history of prior strokes (and related treatment)), other cardiovascular illness (e.g., atherosclerosis and atrial fibrillation), neuroradiology images (e.g., magnetic resonance imaging and computed tomography), and death, if applicable. Individuals were considered diabetic if their fasting blood glucose exceeded 8 mmol/L and their HbA1c exceeded 6.5%. Hyperlipidemia was diagnosed if blood LDL-c exceeded 2.6 mmol/L, and hypertension if blood pressure exceeded 140/90 mmHg. To safeguard patient data, all identifying information was replaced with a code, and procedures were followed throughout the process to ensure data safety and anonymity.

The Institutional Review Board of King Abdullah International Medical Research Center and King Saud Bin Abdulaziz University for Health Sciences, Riyadh, Kingdom of Saudi Arabia (IRB number: SP20/390/R) approved the study. Because this was a retrospective study, informed consent of patients was not required, and the ethics committee waived it. During data entry, each participant received a code so that names and identities were kept secret throughout data administration and analysis.

Statistical analysis

The statistical tool JMP (JMP Inc., Cary, NC, USA) was used for data entry and analysis. Clinical characteristics (e.g., diabetes, hypertension, smoking, valvular heart disease, history of transient ischemic attack or stroke, atrial fibrillation, coronary artery disease, hyperlipidemia, and alcohol use) are presented as frequencies and percentages, whereas numerical data such as age are presented as means and standard deviations (SDs). Correlations between comorbidities and the output (i.e., ICS) were assessed using regression and Pearson’s chi-squared test. For data management and analysis, the biostatistician blindly analyzed the data and did not participate in data collection, which was conducted by other members. To further minimize bias, the neurologist did not participate in the data management process.

## Results

Between 2015 and 2020, 1,870 (65.1% males) patients were admitted to the KAMC neurology department for stroke. The average age was 58.6 ± 13.87 years, and the average BMI was 29.7 ± 6.2 kg/m^2^ (Table 1). Comorbidities included hypertension (66.1%), diabetes mellitus (DM) (57.7%), hyperlipidemia (28.4%), and stroke history (22.3%). Smoking (14.5%), coronary heart disease (9.8%), valvular heart disease (2.9%), atrial fibrillation (5.5%), and transient ischemic attack (3%) were less common (Table 2).

**Table 1:** Descriptive data of all stroke patients SD: standard deviation, BMI: body mass index

		Number	Mean ± SD
Age		1,870	58.6 ± 13.87
Pulse rate		1,827	86.5 ± 16.3
Weight (kg)		1,805	79.1 ± 15.2
Height (cm)		1,800	163.6 ± 9.1
BMI		1,789	29.7 ± 6.2
Gender		Number	%
	Males	1,219	65.2%
	Females	651	34.8%
Total		1,870	

**Table 2:** Comorbidities of all stroke patients DM: diabetes mellitus, VHD: valvular heart disease, TIA: transient ischemic attack, AF: atrial fibrillation, CHD: coronary heart disease

Comorbidity	Number	%
DM	1,079	57.7%
Hyperlipidemia	531	28.4%
Hypertension	1,237	66.1%
Smoking	272	14.5%
Old stroke	417	22.3%
VHD	54	2.9%
Old TIA	56	3%
AF	102	5.5%
CHD	184	9.8%

Compared to ICH stroke (n = 103, 5.5%), ischemic stroke (n = 1,767, 94.5%) was more prevalent (95% confidence interval (CI): 4.5%-6.6%) (Table 3). After accounting for the uneven sample size, both ischemic and ICH strokes increased progressively, but not significantly (p = 0.534), among patients aged 40-70 years. Ischemic stroke prevalence was lowest in patients under 40 years old (10.9%), followed by 15.1%, 24.9%, and 28.3% in patients aged 41-50, 51-60, and 61-70 years old, respectively. Similarly, among patients with ICH stroke, 10.6%, 20.3%, 24.2%, and 28.1% occurred among patients aged <40, 41-50, 51-60, and 61-70 years, respectively. Among patients over 71 years old, the prevalence of both ischemic and ICH strokes reduced to 23.3% and 16.5% in their respective stroke groups (Table 4).

**Table 3:** Distribution of intracerebral hemorrhagic and ischemic stroke among patients ICH: intracerebral hemorrhage

Type of stroke	Number	%
ICH	103	5.5%
Ischemic stroke	1,767	94.5%
Total	1,870	100%

**Table 4:** Prevalence of intracerebral hemorrhagic stroke based on age group and gender (total cases = 1,870) p < 0.05 is considered significant.

		Number	% of all cases	% of the total number	p-value
Age group	≤40	11	0.6%	10.6%	0.534
	41-50	21	1.1%	20.3%	
	51-60	25	1.3%	24.2%	
	61-70	29	1.6%	28.1%	
	71+	17	0.9%	16.5%	
	Total	103	5.5%	100%	
Gender	Female	22	1.17%	21.3%	0.003
	Male	81	4.33%	78.7%	
	M/F ratio	3.7	3.7	3.7	
	Total	103		100%	

No significant age difference was observed in the incidence of ICH stroke (p < 0.534). However, both stroke types were more prevalent and significant in male patients than in female patients (p = 0.003). Out of 1,767 in the IS group, 1,138 were male and 629 were female (64.5% versus 35.5%). Out of 103 patients in the ICH group, 81 were male and 22 were female (78.7% versus 21.2%) (Table 4).

The prevalence of all stroke types varied depending on patients’ BMI, but the total prevalence did not change substantially with increasing BMI (p = 0.081). In the ICH group, of all stroke cases, 0.2% (n = 3) were underweight, 1.3% (n = 24) were normal, 2.2% (n = 42) were overweight, and 1.6% (n = 30) were obese, and of all ICH cases, 3% were underweight, 24.2% were normal, 42.4% were overweight, and 30.3% were obese (Table 5).

**Table 5:** Prevalence of intracerebral hemorrhagic stroke based on BMI p < 0.05 is considered significant. BMI: body mass index

		Number	% of the total cases	% of all ICH cases	p-value
BMI	Underweight	3	0.16%	3%	0.081
	Normal	24	1.3%	24.2%	
	Overweight	42	2.2%	42.4%	
	Obese	30	1.6%	30.3%	
	Total	99	5.26	100%	

Fasting blood glucose > 8 mmol/L (p = 0.032), HbA1c > 6.5% (p = 0.032), hypertension ≥ 140/90 mmHg (p = 0.01), and LDL-c > 2.6 mmol/L (p = 0.002) were significantly linked to ICH stroke in 75%, 48%, and 14% of patients, respectively. However, no significant relation was observed between ICH stroke and a history of previous stroke, smoking, coronary heart disease, valvular heart disease, atrial fibrillation, or previous transient ischemic attack (p > 0.5) (Table 6).

**Table 6:** Percentages of various comorbidities among patients with intracerebral hemorrhage p < 0.05 is considered significant. ICH: intracerebral hemorrhage, VHD: valvular heart disease, TIA: transient ischemic attack, AF: atrial fibrillation, CHD: coronary heart disease, DM: diabetes mellitus

ICH				
		Number	%	p-value
VHD	No	99	99%	0.363
	Yes	1	1%	
Old TIA	No	100	100%	0.997
	Yes	0	0%	
AF	No	99	99%	0.091
	Yes	1	1%	
CHD	No	94	94%	0.229
	Yes	6	6%	
Smoking	No	87	87%	0.362
	Yes	13	13%	
Hyperlipidemia	No	86	86%	0.002
	Yes	14	14%	
Hypertension	No	25	25%	<0.001
	Yes	75	75%	
DM	No	52	52%	0.032
	Yes	48	48%	
Old stroke	No	83	83%	0.186
	Yes	17	17%	

Furthermore, regression analyses showed that only hyperlipidemia (p = 0.066, exponential regression (EXP) = 0.431, 95% CI: 0.409-1.037) and hypertension (p = 0.001, exponential regression (EXP) = 2.453, 95% CI: 1.432-4.202) were substantially linked to ICH stroke. Diabetes mellitus had a nonsignificant tendency to lower the risk of ICH stroke (p = 0.071, exponential regression (EXP) = 0.651) (Table 7).

**Table 7:** Regression analysis of all risk factors with the incidence of intracerebral hemorrhage among the study population p < 0.05 is considered significant. EXP: exponential regression, CI: confidence interval, DM: diabetes mellitus, AF: atrial fibrillation, VHD: valvular heart disease, TIA: transient ischemic attack, BMI: body mass index

	p-value	EXP	95% CI for EXP	
			Lower	
Age group	0.637			
≤40	0.772	0.887	0.396	1.991
40-50	0.751	1.14	0.508	2.556
51-60	0.893	0.947	0.426	2.101
60-70	0.897	1.055	0.471	2.359
70+	0.376	0.678	0.286	1.604
Gender	0.011	1.954	1.164	3.278
Hypertension	0.001	2.453	1.432	4.202
DM	0.071	0.651	0.409	1.037
Hyperlipidemia	0.006	0.431	0.236	0.786
Coronary artery disease	0.407	0.693	0.291	1.649
Smoking	0.113	0.599	0.318	1.128
AF	0.159	0.234	0.031	1.765
VHD	0.546	0.535	0.07	4.082
Old stroke	0.134	0.655	0.376	1.139
Old TIA	0.997	0	0	
BMI	0.263			
Underweight	0.199	2.452	0.483	8.113
Normal	0.297	0.492	0.13	1.865
Overweight	0.195	0.424	0.116	1.554
Obese	0.098	0.33	0.088	1.227

Aspirin, statins, warfarin, and Plavix therapies seemed to confer protection against ICH stroke, as indicated by the relatively low numbers of patients in the ICH stroke group (86, 72, 98, and 87, respectively) who did not receive these medicines at the time of admission (p < 0.05). ICH stroke occurred in 60 patients who were taking combination antihypertensive medication such as angiotensin-converting enzyme (ACE) inhibitors, β-blockers, and Ca2+- channel blockers (p < 0.05), whereas ICH stroke occurred in only 10 patients who were not taking any antihypertensive therapy (p < 0.001) (Table 8).

**Table 8:** Prevalence of intracerebral hemorrhage based on the type of medication taken by patients p < 0.05 is considered significant.

		Number	Ratio	p-value
Aspirin	No	86	86%	<0.001
	Yes	14	14%	
Warfarin	No	98	98%	0.039
	Yes	2	2%	
Plavix	No	87	87%	<0.001
	Yes	13	13%	
Statins	Yes	28	28%	<0.05
	No	72	72%	

## Discussion

This study aimed to identify the prevalence of ICH stroke and common physiological and pathophysiological risk factors in a Saudi population. The findings indicate a relatively low rate of ICH stroke (5.5%), particularly in comparison to older reports in the Middle East region showing rates of 7%-41.5% [12,13] and a significant rise in the prevalence of IS (94.5%). Being male and having hypertension are two main variables linked to an elevated risk of ICH stroke, whereas hyperlipidemia and a high LDL-c ratio seem to confer some protection against ICH stroke. Educating patients about hypertension and how to manage it is critical to reducing the occurrence of strokes. A recent cross-sectional study in Saudi Arabia shows that patients understand the importance of maintaining low blood pressure [19], which might explain the low prevalence of ICH stroke observed in the current study, although such data were not collected or assessed.

Along with comorbidities such as diabetes and hypertension, which alter the size and microvasculature of the brain, the risk of ICH stroke increases with age [20]. Various demographic factors also can have a substantial impact on the relationship between age and ICH stroke [1]. One study found that the risk of stroke starts to increase at age 40 and peaks at ages 51-60 [21]. The current study similarly found that the average age of patients admitted for ICH stroke was 58.6 ± 13.8 years. Furthermore, the rate of ICH stroke increased among patients aged 40-70 years and then decreased in those over 75 years. Despite these findings, increasing age did not have a larger effect on ICH stroke in these older groups, which might indicate that older patients adhere to their antihypertensive medication regimens.

Similar to age, gender also plays a role in the development of ICH stroke. Epidemiological and large-scale meta-analysis studies have shown that males are more likely than females to experience an ICH stroke. This difference has been attributed to lower blood pressure among women throughout their lives [22]. Surprisingly, gender also has an age-dependent influence on the prevalence of ICH stroke. Males have a higher risk than females before the age of 80, after which females have a higher risk [11]. Other studies have similarly found that males have a higher risk of ICH stroke than females up to the age of 75 years [20,23]. In the current study, most participants were under 70 (58.6 ± 13.87) years, and ICH rates were significantly higher in males than in females (odds ratio = 1.954). This result is consistent with prior Saudi Arabian investigations and demographic studies. For example, a hospital-based study of 243 patients with ICH stroke aged 56.9 ± 18.2 years revealed a close ratio of 2.74 [21]. In addition, a meta-analysis of ICH stroke in the general population revealed a higher risk in males (odds ratio = 1.35) [24]. Another systemic review found that the age-adjusted risk of ICH stroke was greater in males than in females between the ages of 65 and 74 years, with a ratio of 1.74 [25].

An important finding in this study is the robust link between hyperlipidemia (LDL-c > 2.6 mmol/L) and the risk of ICH stroke. Only 14% of ICH stroke patients had elevated LDL-c values, suggesting that hyperlipidemia offers some protection in this population. These findings support several epidemiological and meta-analysis studies showing that hyperlipidemia doubles the risk of IS compared to ICH stroke, owing to atherosclerosis [26]. In addition, hypercholesterolemia is negatively associated with ICH stroke [24]. Another fascinating finding of this study is that, in contrast to the 28% of ICH stroke patients who received atorvastatin, simvastatin, or rosuvastatin, 72% never received statin therapy, not even in the hospital. These findings suggest that statins may have no role in the low incidence of ICH stroke among our patients. The impact of lipid-lowering drugs (e.g., statins) on the risk of ICH stroke is still controversial and warrants further research. Some epidemiological studies have shown that statin use increases the risk of ICH stroke with age [27]. Other clinical studies found that statins have no effect on the risk of ICH stroke but reduce the risk of IS [6,28]. This gene-drug interaction may relate to the apolipoprotein E (APOE2/4 or 4/4) mutation, which may increase the chance of developing statin-related lobar ICH stroke [6,10].

The findings demonstrate no link between BMI and the risk of ICH stroke. We observed a nonsignificant escalation in the incidence of ICH stroke when BMI changed. Previous research has found an ambiguous link between BMI and ICH stroke risk, showing that a higher BMI is linked with an increased risk of ICH stroke due to modifying blood pressure therapy [2]. Obesity also increases the risk of IS due to comorbidities such as diabetes and atherosclerosis [20,29].

According to the worldwide INTERSTROKE research and many other epidemiological studies, hypertension is the most common cause of ICH stroke, accounting for up to 83% of cases [2], and it was detected in 75% of our participants. Furthermore, our regression data revealed that the risk of developing an ICH stroke due to hypertension is 2.45. This percentage is lower than what has been documented for ICH stroke patients in high-income Western and Asian countries. For example, a systematic review done by Ariesen et al. found that the crude odds ratio for the connection between ICH stroke and hypertension was 3.68 [24], and the ratio in the USA is 5.5 [29]. This discrepancy between the current research and prior data might be explained by patients’ increasing awareness and dedication to hypertension prevention and treatment [19].

The connection between diabetes and ICH is debatable. The prevalence of diabetes was 48% among the ICH stroke patients in this research, which is higher than previously reported by the INTERSTOKE research and other epidemiological studies (<10%) [2]. In addition, we observed a nonsignificant tendency in our study population for diabetes to reduce the risk of ICH stroke, which aligns with other research showing no link between diabetes and the risk of ICH stroke [26,29].

Antiplatelet and antithrombotic medicines may increase the risk of ICH stroke and brain hemorrhage. Patients using anticoagulants (e.g., aspirin and warfarin) are 7-10 times more likely to have an ICH stroke than those who do not, with up to 20%-30% of all ICH stroke cases falling into this category [30]. Most (>86%) ICH stroke patients in our research were not on any antiplatelet medication, a significant observation that might explain the low frequency of ICH stroke in the study sample.

Despite these intriguing findings, this study does have several significant limitations. First, the data came from hospital-based research based on a single population. Second, the sample size is not sufficiently large enough to represent the entire Saudi population. Third, the information was gathered from individuals who were admitted to KAMC and did not include additional patients who may have been treated at private hospitals. To validate the results and establish the prevalence of ICH stroke in the Saudi population, further large-scale investigations covering the main hospitals in Riyadh and other locations are needed. Fourth, the study might be skewed by the quality of the medical records and data collected from patients. Fifth, the study did not quantify various social, socioeconomic, stress, physical activity, and healthcare system factors that could affect ICH stroke. Future research should take this into account. These limitations and the interesting results presented should guide future researchers and clinicians for better research design and clinical practice.

## Conclusions

The current study’s findings showed that males have a higher risk of stroke than females and that ICH stroke increases progressively, but not significantly, with age from 40 to 70 years. The risk of stroke was highest in the 61-70 years age group. The result showed that the most significant factors elevating the risk of developing ICH were hypertension (75%), diabetes (48%), and hyperlipidemia (14%). Hypertension also had a positive association with the progression of ICH, whereas hyperlipidemia had an inverse association. This study is implying that rates of ICH stroke are declining in the study population; however, a longitudinal study design is a better option to confirm this statement.
